# Abortion Statistics

**Published:** 1898-02

**Authors:** 


### Abortion Statistics.

Dr. C. D. Arnold, of El Reno, Oklahoma, wishes answers to the following questions from every physician who reads them:

1. Give total number of abortion from all causes that occurred in your practice during 1897?

2. In how many of these abortions were the elements of criminality, to your mind, apparent?

3. In how many of these abortions, except those classed in question 2, were the elements of criminality, to your mind probable?

4. How many of the abortions named in question 2 and 3 were followed by puerperal septicaemia or other diseases?

5. How many deaths resulted from the abortions named in questions 2 and 3?

6. How many still-born in your practice?

7. How many infanticides?

8. How many viable children born in your practice?

9. How many cases of puerperal mania resulted from the abortions classed in questions 2 and 3?

